# Reply to editorial “From Probability to Practice: Demystifying When Lung Shunt Fraction Matters in ^90^Y Selective Internal Radiation Therapy”

**DOI:** 10.1093/radadv/umag010

**Published:** 2026-02-26

**Authors:** M Allan Thomas, Christopher D Malone

**Affiliations:** Mallinckrodt Institute of Radiology, Washington University School of Medicine, St. Louis, MO, 63130, United States; Mallinckrodt Institute of Radiology, Washington University School of Medicine, St. Louis, MO, 63130, United States

**Keywords:** lung shunt fraction, lung dose, ^90^Y, radioembolization, selective internal radiation therapy, radiation segmentectomy, macroaggregated albumin single photon emission computed tomography/CT, streamlined “no-MAA” ^90^Y

We appreciated the favorable commentary on our recent article^1^ by Li et al.^2^ Nevertheless, we would like to offer some points of note. Our focus on whole-liver dose thresholds and whole-liver volume instead of perfused volume (PV) and dose may seem misaligned with modern treatment planning. However, we aimed to develop a method employing case-specific parameters that would be readily available for the largest number of patients. Whole liver volume will always be known prospectively, while PV estimation before any angiographic procedures remains speculative. Redefining the boundary lung shunt fraction (LSF_bound_) metric in terms of PV and PV dose, as outlined by Li et al., may seem intuitive, but this approach explicitly requires measured or estimated PV. We contend this will amplify uncertainty and reduce clinical applicability, rather than simplify our original framework in a meaningful way.

The other simplification, a nominal 1-kg lung mass instead of a patient-specific lung mass, also raises concerns. This approach mirrors historical ^90^Y-SIRT treatment planning with generalized assumptions for all patients. Instead, patient-specific data can often be easily acquired and employed.^3^^,^^4^ The use of 1-kg lung mass for most or all patients will produce *non-linear changes in computed LSF_bound_* that may affect appropriate patient triage for LSF assessment. There would be a particular risk for patients with a lung mass well below 1 kg. Such patients cannot always be identified through other clinical factors.

Finally, applying the original or modified LSF_bound_ method to new clinical data, as outlined by Li et al., was not especially informative. The cases presented were from a “no-MAA” workflow, maintaining clinical characteristics (small HCC, small PV) associated with minimal risk for high LSF.^5^ The observation that the LSF_bound_ approach worked ideally for such cases, with essentially no risk of inappropriate triage for LSF assessment, was predictable. While reassuring, these findings do not substantially extend the scope of validation beyond what would be anticipated for such cases. We instead encourage the field to consider a prospective application of the LSF_bound_ approach across the full spectrum of ^90^Y-SIRT eligible patients at their institution, if deemed appropriate and of interest.
